# Structural and Biochemical Features of *Eimeria tenella* Dihydroorotate Dehydrogenase, a Potential Drug Target

**DOI:** 10.3390/genes11121468

**Published:** 2020-12-07

**Authors:** Dan Sato, Endah Dwi Hartuti, Daniel Ken Inaoka, Takaya Sakura, Eri Amalia, Madoka Nagahama, Yukina Yoshioka, Naotoshi Tsuji, Tomoyoshi Nozaki, Kiyoshi Kita, Shigeharu Harada, Makoto Matsubayashi, Tomoo Shiba

**Affiliations:** 1 Department of Applied Biology, Graduate School of Science Technology, Kyoto Institute of Technology, Matsugasaki, Sakyo-ku, Kyoto 606-8585, Japan; dsatodarby0415@hotmail.co.jp (D.S.); qp.m.324@gmail.com (M.N.); yosioka_y02@yahoo.co.jp (Y.Y.); haradas2919@gmail.com (S.H.); 2 Department of Parasitology, Institute of Tropical Medicine (NEKKEN), Nagasaki University, 1-12-4 Sakamoto, Nagasaki 852-8523, Japan; endah.dwi08@yahoo.co.id; 3 Department of Molecular Infection Dynamics, Institute of Tropical Medicine (NEKKEN), Nagasaki University, 1-12-4 Sakamoto, Nagasaki 852-8523, Japan; takaya.sakura@nagasaki-u.ac.jp; 4 School of Tropical Medicine and Global Health, Nagasaki University, 1-12-4 Sakamoto, Nagasaki 852-8523, Japan; kitak@nagasaki-u.ac.jp; 5 Department of Biomedical Chemistry, Graduate School of Medicine, The University of Tokyo, 7-3-1 Hongo, Bunkyo-ku, Tokyo 113-0033, Japan; amalia_eri@yahoo.co.id (E.A.); nozaki@m.u-tokyo.ac.jp (T.N.); 6 Department of Parasitology, Kitasato University School of Medicine, 1-15-1 Kitasato, Minami-ku, Sagamihara, Kanagawa 252-0374, Japan; tsujin@med.kitasato-u.ac.jp; 7 Department of Host-Defense Biochemistry, Institute of Tropical Medicine (NEKKEN), Nagasaki University, 1-12-4 Sakamoto, Nagasaki 852-8523, Japan; 8 Division of Veterinary Science, Graduate School of Life and Environmental Sciences, Osaka Prefecture University, 1-58 Rinku Orai Kita, Izumisano, Osaka 598-8531, Japan; matsubayashi@vet.osakafu-u.ac.jp

**Keywords:** coccidium, apicomplexa, *Eimeria tenella*, membrane protein, mitochondria, electron transport chain, dihydroorotate dehydrogenase, crystal structure, ubiquinone, inhibitor

## Abstract

Dihydroorotate dehydrogenase (DHODH) is a mitochondrial monotopic membrane protein that plays an essential role in the pyrimidine de novo biosynthesis and electron transport chain pathways. In *Eimeria tenella*, an intracellular apicomplexan parasite that causes the most severe form of chicken coccidiosis, the activity of pyrimidine salvage pathway at the intracellular stage is negligible and it relies on the pyrimidine de novo biosynthesis pathway. Therefore, the enzymes of the de novo pathway are considered potential drug target candidates for the design of compounds with activity against this parasite. Although, DHODHs from *E. tenella* (EtDHODH), *Plasmodium falciparum* (PfDHODH), and human (HsDHODH) show distinct sensitivities to classical DHODH inhibitors, in this paper, we identify ferulenol as a potent inhibitor of both EtDHODH and HsDHODH. Additionally, we report the crystal structures of EtDHODH and HsDHODH in the absence and presence of ferulenol. Comparison of these enzymes showed that despite similar overall structures, the EtDHODH has a long insertion in the N-terminal helix region that assumes a disordered configuration. In addition, the crystal structures revealed that the ferulenol binding pocket of EtDHODH is larger than that of HsDHODH. These differences can be explored to accelerate structure-based design of inhibitors specifically targeting EtDHODH.

## 1. Introduction

Poultry industries are among the most important animal food resources, and their global production has tripled in a quarter-century (FAO, 2015). Hence, pathogens infecting chickens pose serious threats to world food security because such pathogens lead to reduced productivity. In particular, poultry coccidiosis has the greatest economic impact on productivity because of high infectivity and mortality [1], with an estimated annual worldwide economic loss of 2 billion dollars. More than 1200 species of *Eimeria* are known; these species typically have been defined based on host specificity [2]. Amongst the seven *Eimeria* species that are known to infect chickens, *Eimeria tenella* infection has the greatest impact in global agro-economics due to this pathogen’s high virulence [3].

In currently used preventive therapies, vaccines with attenuated pathogens are encouraged due to the rise of drug resistance and public concerns for residue-free meat [3]. Although, the immune strategy can reduce the severity of diseases, it does not inhibit the infections, resulting in oocyst shedding in feces as potential sources of new infection and spread [3]. Hence, the development of new prophylactic modalities is indispensable for satisfactory prevention (or eradication) of the disease, e.g., suppressing the infections and possessing non-toxic and protozoan-specific effects.

Pathogen-specific mitochondrial pathways are validated drug targets. Such pathways have been explored for the development of new drugs against malaria, a disease that is caused by several *Plasmodium* parasites belonging to the phylum Apicomplexa. For example, the antimalarial drug atovaquone targets the *Plasmodium falciparum* mitochondrial electron transport chain (ETC) by binding at the *Q*_o_ site of the cytochrome *b* subunit of the *bc*_1_ complex [4,5,6]. Inhibition of the *bc*_1_ complex results, not only in the inhibition of oxygen respiration, but also the inhibition of all upstream ETC dehydrogenases, which comprise the non-proton-motive type II NADH dehydrogenase (NDH-2), succinate dehydrogenase (complex II), malate:quinone oxidoreductase (MQO), glycerol-3-phosphate dehydrogenase (G3PDH), and dihydroorotate dehydrogenase (DHODH) [7,8]. Complex II and MQO are components of the TCA cycle, and NDH-2 is required to re-oxidize NADH produced in mitochondrial metabolism, including the TCA cycle. Genetic validation conducted using *P. berghei*-infected mice or cultured *P. falciparum* has shown that NDH-2 [9,10] and complex II [11,12] are essential for oocyst development inside the mosquito, while MQO is required (at minimum) in the asexual blood stage [7,13]. Little is known about the role of G3PDH in apicomplexan parasites, although some studies have suggested a role for G3PDH in redox-balance, gluconeogenesis, and lipid biosynthesis [14,15]. DHODH catalyzes the oxidation of dihydroorotate and the reduction of an acceptor in the fourth and rate-limiting step of de novo biosynthesis of pyrimidines, molecules that serve as building blocks for DNA and RNA and are indispensable metabolites in all living organisms [16,17]. Animals commonly utilize two pathways for the biosynthesis of pyrimidines, de novo and salvage pathways. As also seen for *Toxoplasma gondii*, the genes encoding enzymes for pyrimidine de novo pathways seem to be conserved in the genome of *E. tenella*. Although a gene encoding uracil phosphoribosyltransferase (accession: AET50663), a key pyrimidine salvage pathway enzyme, can be found in the genome of *E. tenella*, the activity of this pathway appears to be negligible, at least in the intracellular stage, making this pathogen dependent on the de novo pathway to meet the cellular demand for pyrimidines [18,19,20]. Therefore, the enzymes of the de novo pathway are potential drug target candidates for the development of new compounds with activities against coccidian parasites.

Based on localization and electron acceptor, DHODHs are classified into two families: Family 1 members are soluble proteins localized to the cytosol, while family 2 members are membrane proteins localized to the inner mitochondrial membrane. Family 1 is further subdivided into family 1A and family 1B, which use fumarate and NAD^+^ (respectively) as electron acceptors [17,21,22]. Family 2 DHODH enzymes use respiratory quinones as electron acceptors [17,22,23,24]. The anti-proliferative agent leflunomide targets the quinone binding site of human DHODH (HsDHODH) and is used for the treatment of rheumatoid arthritis [25]. We recently reported that ascofuranone, an antibiotic isolated from *Acremonium egiptiacum* [26], and its synthetic derivatives are potent inhibitors of HsDHODH and exhibit selective cytotoxicity against cancer cells grown under conditions mimicking the tumor microenvironment (hypoxia and nutrient deprivation) [17]. *Helicobacter pylori*- and *P. falciparum*-specific DHODH inhibitors have been developed, and include compounds, such as intervenolin derivatives [23], and DSM265 [27], respectively. Recently, data from the Phase IIa trial of DSM265 indicated satisfactory results against the blood and liver stages of *P. falciparum*, suggesting the potential utility of this compound for the treatment and prevention of infection by this pathogen [27,28,29].

For structure-based drug design, the three-dimensional structure of a target protein is required. To our knowledge, the X-ray crystal structures of DHODHs, ligand-free and/or in complex with inhibitors, have been reported for human [17,30], rat [31,32], *Escherichia coli* [33], *Streptococcus* sp. *mutans* [34], *Mycobacterium tuberculosis* (PDB entry 4XQ6), *Trypanosoma cruzi* [16,22], *T. brucei brucei* [35,36], *Leishmania donovani* (PDB entry 3C61), and *P. falciparum* [37]. However, a potent inhibitor of family 2 DHODH from any livestock parasite, and the crystal structure of the corresponding enzyme, have yet to be determined.

In this report, as a contribution toward the structure-based drug design of a new class of anti-coccidial agents, we have purified recombinant *E. tenella* DHODH (EtDHODH) and identified the enzyme’s first nanomolar inhibitor. Moreover, the crystal structure of EtDHODH was determined in the absence and presence of the inhibitor. Comparative analysis with HsDHODH revealed important differences within the inhibitor binding pocket; such differences can be explored to design potential selective drugs for prevention of chicken coccidiosis.

## 2. Materials and Methods

### 2.1. Sequence Analysis of E. tenella DHODH

The protein sequences of DHODHs were analyzed using the ClustalW platform and the amino acid percentage identities calculated by the pairwise alignment tool from Jalview 2.9.0b2 software, according to developer’s protocol (Figure S1) [38].

The atomic coordinates and structural factors have been deposited in the Protein Data Bank, www.odb.org (EtDHODH ligand-free form: 6AJ5, EtDHODH-ferulenol complex: 6AJE, HuDHODH ferulenol complex: 6IDJ).

### 2.2. Expression and Purification of EtDHODH, PfDHODH, and HsDHODH

The sequence of the gene encoding a putative DHODH, as annotated in the genomic DNA of *E. tenella* strain Houghton (GenBank accession number CDJ37002), was confirmed by DNA sequencing of a complementary DNA (cDNA) prepared from *E. tenella* sporozoites [39]. Based on the deduced amino acid sequence, we synthesized a full-length of EtDHODH gene that was codon optimized for expression in *E. coli*; the resulting fragment was cloned into the pET19b plasmid vector (Novagen, Madison, WI, USA) via flanking NdeI and BamHI sites using a TaKaRa In-Fusion HD Cloning Kit (TaKaRa, Kyoto, Japan). The resulting plasmid (pET19b/EtDHODH) was transformed into the *E. coli* BL21 Star™ (DE3) strain (Invitrogen, Carlsbad, CA, USA) for protein expression. The transformant was grown in Luria–Bertani (LB) medium containing 50 μg/mL carbenicillin and 1% (*w/v*) glucose at 37 °C. The pre-culture (30 mL) was inoculated into 1 L of LB medium containing carbenicillin. When this culture reached the mid-log growth phase (optical density at 660 nm (OD660) = 0.5), expression of the recombinant protein was induced by the addition of 1 mM isopropyl *β*-D-thiogalactopyranoside (IPTG) and 20 μM riboflavin at 18 °C for 18 h. The cells were harvested by centrifugation at 8000× *g* for 20 min and stored at −80 °C until use. The bacterial pellets collected from 6 L of culture were suspended in 40 mL of Buffer A (50 mM HEPES, pH 7.6, containing 300 mM NaCl, 10% (*v/v*) glycerol, 0.2 mM sodium orotate) with complete mini EDTA-free Protease inhibitor cocktail (Roche, Basel, Switzerland). The suspension was passed through a French Pressure Cell three times at 200 MPa to lyse the cells. The lysate was centrifuged at 20,000× *g* (4 °C) to remove unbroken cells and inclusion bodies. The supernatant, which contained suspended membrane particles, was mixed with Triton X-100 to a final concentration of 1% (*w/v*), and further centrifuged for 90 min at 40,000× *g* (4 °C) to remove residual undissolved materials. The supernatant was applied to a HisTrap FF column (i.d. 0.7 cm × 2.5 cm, GE Healthcare, Chicago, IL, USA) equilibrated with Buffer A plus 10 mM N,N-dimethylundecylamine N-oxide (UDAO, Santa Cruz Biotechnology, Dallas, TX, USA). After washing the column with the same buffer until the absorbance at 280 nm was negligible, recombinant EtDHODH protein was eluted stepwise with Buffer A containing 20, 100, 300, and 500 mM imidazole. Fractions containing highly purified protein, as assessed by sodium dodecylsulfate polyacrylamide gel electrophoresis (SDS–PAGE), were pooled and the buffer was exchanged to Buffer B (50 mM HEPES, pH 7.6, containing 400 mM NaCl, 30% (*v/v*) glycerol, 0.2 mM sodium orotate, 10 mM UDAO, and 1 mM EDTA) using a centrifugal ultrafiltration device (Amicon Ultra-15, 50-kDa molecular weight cut-off; Merck Millipore, Burlington, MA, USA). The EtDHODH eluted at 300 mM imidazole showed the highest purity (Figure S2) and was used for subsequent analysis. The purified protein was stored in 50% (*v/v*) glycerol at −80 °C until use. Compared to the protein predicted from the genome, the final recombinant EtDHODH protein contained an additional 23 N-terminal amino acid residues (MGHHHHHHHHHHSSGHIDDDDKH) that were derived from the expression vector.

Sequences encoding a truncated (lacking residues 1-157) *P. falciparum* DHODH (PfDHODH) and codon-optimized for *E. coli* expression were inserted into the pETSUMO expression vector to generate pETSUMO/PfDHODH and used to transform BL21 Star™ (DE3). Ten milliliters of pre-culture was used to inoculate into 500 mL of TB medium supplemented with 50 μg/mL kanamycin in an Ultra yield flask and cultured at 37 °C with shaking at 250 rpm. After the OD600 reached 0.4–0.6, PfDHODH expression was induced by addition of 0.2 mM IPTG, and the culture was incubated for a minimum of 16 h at 20 °C with shaking. All of the following purification steps were conducted at 4 °C. The harvested cells were suspended in 20 mL of lysis buffer (50 mM HEPES-KOH, pH 7.6, 500 mM NaCl, 5 mM imidazole, 20% (*v/v*) glycerol, 0.25 mM of phenylmethanesulfonyl fluoride (PMSF)) and broken by French Press at 180 MPa. Unbroken cells and debris were removed by centrifugation at 25,000× *g* for 20 min to obtain the extract. Triton X-100 was added to the extract to a final concentration of 1% (*w/v*); the mixture was stirred for 30 min and further centrifuged at 200,000× *g* for 90 min. The resulting supernatant was mixed with 13 mL of Ni-NTA resin (Qiagen, Hilden, Germany) and rotated for 1 h at 10 rpm. After centrifugation at 1500× *g* for 15 min, the supernatant was discarded; the resin pellet was suspended in a minimal volume of supernatant and packed into a gravity-flow column. The column was washed consecutively with 60 mL of Buffer A (50 mM HEPES-KOH, pH 7.5, 300 mM NaCl, 10% (*v/v*) glycerol, 0.2 mM orotate), 60 mL of Buffer A containing 0.05% (*w/v*) C_12_E_9_, and 60 mL of Buffer A containing 5 mM imidazole and 0.05% (*w/v*) C_12_E_9_. Bound His6-SUMO-PfDHODH was eluted using 30 mL of Buffer A containing 300 mM imidazole and 0.05% (*w/v*) C_12_E_9_, and the eluate was collected at 1 mL per fraction. The protein content of each fraction was monitored at 280 nm using a Nanodrop spectrophotometer. The protein-containing fractions were pooled and concentrated using an Ultracel 50 kDa MWCO device at 3500× *g* until the volume fell below 0.5 mL. Buffer A was added to the device eluate to yield a final volume of 15 mL, and the mixture was subjected to another round of concentration to reduce the imidazole concentration. To remove the His6-SUMO tag, one-twentieth amount of SUMO protease was added to the PfDHODH. The mixture was diluted with cleavage buffer (50 mM Tris HCl pH 8.0, 300 mM NaCl, 0.05% (*w/v*) C_12_E_9_, 0.2 mM orotate) and incubated for 14.5 h. Purification of His6-SUMO-tag-free native PfDHODH was achieved by loading the digestion mixture onto a 13-mL Ni-NTA column and collecting the flow-through. Finally, the purified PfDHODH was concentrated using an Ultracel 50 kDa MWCO device at 3500× *g*. Ice-cold glycerol was added to the eluate to a final concentration of 50% (*v/v*), and this glycerol stock of purified PfDHODH was stored at −30 °C until use. The purity of PfDHODH at each step of purification was evaluated by electrophoresis of aliquots on a SDS-PAGE.

A gene encoding HsDHODH, without its mitochondrial targeting signal (residues 30-396), was inserted into pET19b at the BamHI and NdeI sites. The resulting plasmid was used to transform the BL21(DE3)Δ*PyrD E. coli* strain, and the enzyme was purified as described previously [17,22]. Briefly, a pre-culture of BL21(DE3)Δ*PyrD*/pET19bHsDHODH was prepared in 800 mL of 2 × YT medium supplemented with 100 µg/mL of carbenicillin and 50 µg/mL of kanamycin, and incubated with shaking (200 rpm) at 25 °C overnight. The pre-culture was centrifuged at 3000× *g* for 15 min at room temperature and the resulting pellet resuspended in 400 mL of fresh 2 × YT medium supplemented with same concentrations of antibiotics. This *E. coli* suspension was inoculated into 7.8 L of 2 × YT prepared in a fermentor jar and used as the main culture. To avoid excessive foaming, 5 mL of antifoam (FS antifoam DB-110, Dow Corning, Midland, MI, USA) was added to the main culture, which was then incubated at 25 °C, with mixing at 600 rpm and aeration at Level 7. When the OD600, which was monitored hourly, reached 1.0–1.2 (approximately 2 h), IPTG was added to a final concentration of 1 µM. The culture then was further incubated for 16 h at 25 °C, with mixing at 600 rpm and aeration Level 7. All subsequent procedures were performed at 4 °C. Cells were harvested by centrifugation at 4000× *g* for 15 min, and the resulting pellet was suspended to a final volume of 210 mL in Buffer A (50 mM HEPES-KOH, pH 7.6, 300 mM NaCl, 10% (*v/v*) glycerol, 0.2 mM orotate) and broken by single passage in a French Press at 180 MPa. The resulting lysate was centrifuged at 20,000× *g* for 10 min to obtain the extract. Triton X-100 was added into the extract to a final concentration of 1% (*w/v*), and the mixture was stirred for 30 min before centrifugation at 200,000× *g* for 75 min. The resulting supernatant containing solubilized HsDHODH was combined with 10 mL of Talon^®^ Superflow metal affinity resin (Clontech, Mountain view, CA, USA) pre-equilibrated with Buffer A and mixed for 1 h. The protein-resin mixture was loaded onto a column, the flow-through fractions collected, and the resin washed consecutively with 100 mL of Buffer A and 200 mL Buffer A containing 10 mM UDAO. The purified protein was eluted by application of a stepwise gradient of 16, 30, and 160 mM imidazole in Buffer A containing 10 mM UDAO. The yellow active fractions were pooled and concentrated to a volume of less than 2 mL using an Ultracel 30 kDa MWCO centrifugal device at 5000× *g*, then filtered using an Ultrafree-CL Low Durapore 0.45-µm membrane. Glycerol added to a final 50% (*v/v*) concentration, and the stock was stored at −30 °C until use.

### 2.3. Enzyme Assays and Protein Concentration Determination

All assays were performed using a Jasco V-660 spectrophotometer connected to a circulator and black quartz cuvettes in total 1 mL reaction volumes.

EtDHODH activity was measured in a reaction mixture containing 100 mM HEPES, pH 7.6, 150 mM NaCl, 5% (*v/v*) glycerol, 0.05% (*w/v*) Triton X-100, 120 μM 2,6-dichlorobenzeneindophenol (DCIP), 14 μM decylubiquinone (dUQ), and purified EtDHODH. After incubation at 37 °C for 5 min, the reaction was initiated by the addition of *L*-dihydroorotate (DHO) to 200 μM, and the decrease in absorbance at 600 nm was monitored. The extinction coefficient of oxidized DCIP_600 nm_ is 21 mM^−1^ cm^−1^.

Routine PfDHODH activity was assayed using an assay solution containing 100 mM HEPES, pH 7.5, 5% (*v/v*) glycerol, 150 mM NaCl, 0.05% (*w/v*) Triton X-100, 120 μM DCIP, 15 μM of dUQ, and the enzyme. The reaction was initiated by the addition of DHO to 200 μM, and the decrease in absorbance at 600 nm was monitored at 25 °C as a measure of the consumption of oxidized DCIP.

HsDHODH activity was measured in an assay mixture containing 50 mM Tris HCl, pH 8.0, 0.1% (*w/v*) Triton X-100, 2 mM KCN, 60 µM dUQ, 120 µM DCIP, and enzyme. The reactions were initiated by the addition of DHO to 500 μM. The decrease in absorbance at 600 nm was monitored at 25 °C as a measure of the consumption of DCIP [17,22].

Protein concentrations were determined by the Lowry method using bovine albumin as a standard [40].

### 2.4. Screening of Inhibitors

A focused library of approximately 200 compounds, comprising known inhibitors of ETC and known antiparasitic agents, was screened against EtDHODH, PfDHODH, and HsDHODH using 96-well plates; compounds were tested in triplicate at concentrations of 2.5 and 10 μM. The screening method was adapted from the previously reported end-point method used for HsDHODH and PfDHODH [17,24].

The assay mix for EtDHODH screening was composed of 100 mM HEPES, pH 8.0, 50 mM NaCl, 10% (*v/v*) glycerol, 0.05% (*w/v*) Triton X-100, 120 μM DCIP, 18 μM dUQ, and 360 ng/mL of purified EtDHODH. First, 193 μL or 194.5 μL of assay mix solution was transferred to each well of a 96-well plate containing (respectively) 2 μL (final 10 μM) or 0.5 μL (final 2.5 μM) per well of 1 mM inhibitor stock solutions (columns 2 to 11). DMSO was added in columns 1 and 12; those columns were used as negative (0% inhibition) and positive controls (100% inhibition, as a result of no substrate added, as noted below), respectively. The plates were mixed well, and background absorbance was recorded for 1 min at 600 nm and 25 °C using a SpectraMax^®^ Paradigm^®^ Multi-Mode Microplate Reader (Molecular Device, San Jose, CA, USA). To initiate the reaction, 5 μL/well of 5 mM DHO was added (except for column 12, the positive control), the plate was mixed well, and the activity recorded for 20 min. End-point measurement of the absorbance was performed after 20 min, and EtDHODH inhibition was determined by calculating the inhibition (%) relative to the activity in the negative and positive control columns. Hits were defined as compounds showing more than 50% inhibition at 2.5 μM.

The screening of inhibitors against PfDHODH was conducted using the same method as for EtDHODH, except that the assay mixture contained 100 mM HEPES, pH 7.5, 5% (*v/v*) glycerol, 150 mM NaCl, 0.05% (*w/v*) Triton X-100, 120 μM DCIP, 15 μM dUQ, and 20 nM of purified PfDHODH. The reactions were initiated by adding DHO to 200 μM. The activity was monitored as for EtDHODH.

The screening of inhibitors against HsDHODH was conducted using the same method as for EtDHODH, except that the assay mixture contained 50 mM Tris-HCl, pH 8.0, 0.1% (*w/v*) Triton X-100, 2 mM KCN, 60 µM dUQ, 120 µM DCIP and 5.85 nM of purified HsDHODH. The reactions were initiated by adding DHO to 500 µM. The activity was monitored as for EtDHODH.

The quality of all screening conducted in this study was monitored by calculating the Z-factor, signal-to-background (S/B), signal-to-noise (S/N), signal window (S/W), and coefficient of variation (CV) for each assay plate, as previously described [41,42].

The ferulenol concentration that reduced the enzymatic activity by 50% (IC_50_) was determined by varying the inhibitor concentration and using a 96-well plate as described above.

### 2.5. Crystallization and X-ray Diffraction Data Collection for EtDHODH

Ligand-free crystals of EtDHODH suitable for X-ray analysis were obtained by the sitting-drop vapor-diffusion method at 20 °C. EtDHODH at a concentration of 13 mg/mL in Buffer A (50 mM HEPES, pH 7.6, 400 mM NaCl, 30% (*v/v*) glycerol, 0.2 mM sodium orotate, 10 mM UDAO, 2% (*w/v*) C_12_E_8_, 1 mM EDTA) was mixed with an equal amount of the reservoir solution (100 mM HEPES, pH 7.0, 1.8 M triammonium citrate) to make a droplet of 0.5 μL and equilibrated against 100 μL reservoir solution. Crystals of EtDHODH-ferulenol complex were prepared by co-crystallization. A 0.5-μL droplet of 11 mg/mL protein solution in Buffer C (10 mM HEPES, pH 7.6, 500 mM NaCl, 30% (*w/v*) glycerol, 0.2 mM orotate, 10 mM UDAO, 2% (*w/v*) C_12_E_8_, 1 mM EDTA, 1 mM ferulenol) was mixed with the same amount of reservoir solution and equilibrated against 100 μL of the reservoir solution. X-ray diffraction experiments were performed on the BL44XU beamline at SPring-8 (Harima, Japan). Diffraction data were processed and scaled with the HKL-2000 software package [43].

### 2.6. Structural Determination of EtDHODH with and without Inhibitor

The initial structure of the inhibitor-free EtDHODH was determined by molecular replacement using the crystal structure of HsDHODH (PDB code: 3W7R), which has 49.6% amino acid identity with EtDHODH, as a search model. The crystal structure of the inhibitor-free EtDHODH was refined at 3.5 Å resolution using REFMAC5 [44] to final *R*_work_/*R*_free_ values of 0.272/0.359 (Table S1). The crystal structure of the EtDHODH-ferulenol complex was determined by molecular replacement using inhibitor-free EtDHODH as a search model. The crystal structure of EtDHODH-ferulenol complex was refined at 3.65 Å resolution to final *R*_work_/*R*_free_ values of 0.221/0.303 (Table S1).

### 2.7. Structure Determination of HsDHODH-Ferulenol Complex

The HsDHODH-ferulenol complex crystals were obtained essentially as described previously [17,22]. X-ray diffraction data of HsDHODH-ferulenol complex crystal was collected at 100 K on the beamline BL-17A (λ = 0.98000 Å; ADSC Quantum 315r, Irving, TX, USA) at Photon Factory (Tsukuba, Japan). The data sets were processed and scaled using HKL-2000 [43]. The crystal structure of the HsDHODH-ferulenol complex was solved by molecular replacement using the refined protein coordinates of HsDHODH-mii-4-087 complex (PDB code 3W7R) [22] as a search model. MOLREP program [45] as implemented within CCP4 package [46] (http://www.ccp4.ac.uk/) was used for molecular replacement. Structural adjustments were made by iterative cycles of manual adjustments in COOT [47] and refinements by REFMAC5 [44]. The crystal structure of HsDHODH-ferulenol complex was refined to final *R*_work_/*R*_free_ values of 0.163/0.178 (Table S1).

## 3. Results

### 3.1. Sequence Analysis of EtDHODH

The alignment as well as the percentage identity of EtDHODH and other family 2 DHODHs are shown in Figure S1. The N-terminal extension found in DHODHs from other apicomplexan parasites, such as *P. falciparum*, *T. gondii*, and *Neospora caninum*, is not conserved in EtDHODH (Figure S1). However, a long insertion from residues R217 to T252 is conserved among the DHODHs from the apicomplexan parasites listed in Figure S1. Moreover, residues essential for interaction with dihydroorotate showed higher levels of conservation than the ones required for interaction with ubiquinone. In addition, EtDHODH showed a higher degree of amino acid identity to mitochondrial DHODHs than to those from bacteria (Figure S1).

### 3.2. Purification of DHODHs

The DHODHs from *E. tenella*, *P. falciparum*, and human used in our study were successfully purified to homogeneity. The method used for purification of EtDHODH was adapted from the reported purification method for HsDHODH using Triton X-100 as the solubilizing detergent. EtDHODH, which was purified in a single step using nickel-affinity chromatography, showed specific activity of 32 μmol/min/mg protein. In the case of PfDHODH, due to the low purification yield by the reported method, we developed a new expression system employing a His6-SUMO tag at the enzyme’s N-terminus. Using this new construct, we purified a SUMO tag-free PfDHODH at yield of 73 mg per liter of culture, with the purified enzyme showing specific activity of 20 μmol/min/mg protein. The HsDHODH, which was purified essentially as described previously [17,22], exhibited a specific activity of 71 μmol/min/mg protein.

### 3.3. Screening for EtDHODH Inhibitors

To identify an inhibitor of EtDHODH, we used a library, obtained from Nagasaki University, containing compounds focused on mitochondrial ETC inhibitors and known antiparasitic agents [41]. The screening method for EtDHODH was adapted from the reported HsDHODH method; we tested potential inhibitors at two concentrations (2.5 and 10 μM). The screening against EtDHODH was shown to be effective, yielding average values for the Z-factor, S/B, S/N, S/W, and CV of 0.866, 1383, 351, 26.0, and 4.02%, respectively. EtDHODH was not inhibited by classical inhibitors of ETC (such as rotenone, atpenin A5, siccanin, flutolanil, antimycin A, stigmatellin, myxothiazol, ascochlorin, and atovaquone) from various organisms, nor by known antiparasitic drugs (such as albendazole, ivermectin, chloroquine, artemisinin, morantel, and nitazoxanide) and potent PfDHODH inhibitor (DSM265). We identified 33 compounds that inhibited EtDHODH activity by more than 50% when tested at concentrations of 2.5 µM. With the exception of ferulenol and its 3 derivatives thereof, the remaining 29 compounds all corresponded to ascofuranone derivatives.

### 3.4. Inhibitor Cross-Sensitivity of EtDHODH, PfDHODH, and HsDHODH

**Figure 1:**
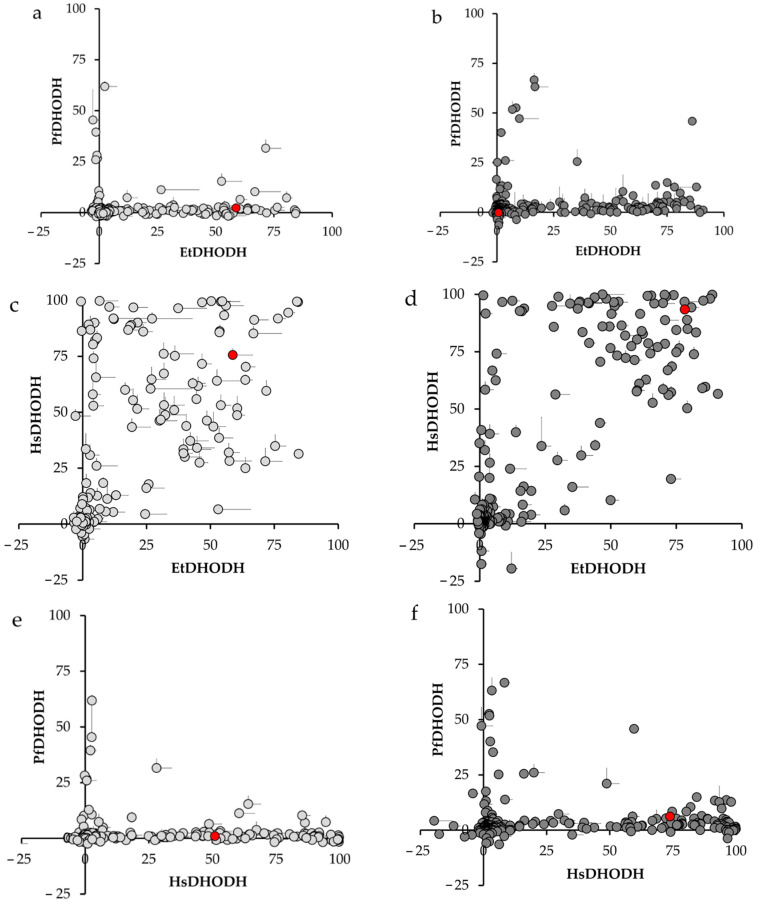
Cross-sensitivity of EtDHODH, PfDHODH, and HsDHODH to inhibitors at final concentrations of 2.5 μM (**a**,**c**, and **e**, respectively) and 10 μM (**b**,**d**, and **f**, respectively). No significant cross-sensitivity was observed between EtDHODH and PfDHODH (**a**,**b**) or between PfDHODH and HsDHODH (**e**,**f**). However, cross-sensitivity was observed between EtDHODH and HsDHODH (**c**,**d**). The position of ferulenol is shown in red. Values plotted represents the average inhibition (*n* = 3). Only the positive value of SD was plotted for better representation.

Next, using the same library, we evaluated the cross-sensitivity of PfDHODH and HsDHODH to the same panel of inhibitors. Figure 1 shows the results of screening at concentrations of 2.5 and 10 μM and compared among EtDHODH, PfDHODH, and HsDHODH. Our results clearly demonstrated that EtDHODH and PfDHODH did not show cross-sensitivity to inhibitors (Figure 1). However, we unexpectedly found that EtDHODH and HsDHODH share cross-sensitivity to two classes of compound: ferulenol, and some of ascofuranone derivatives. Although EtDHODH and HsDHODH showed cross-sensitivity to some of ascofuranone derivatives, there are several inhibitors specific for HsDHODH within this class of compounds (Figure S3).

### 3.5. Ferulenol is a Potent Inhibitor of EtDHODH and HsDHODH

**Figure 2:**
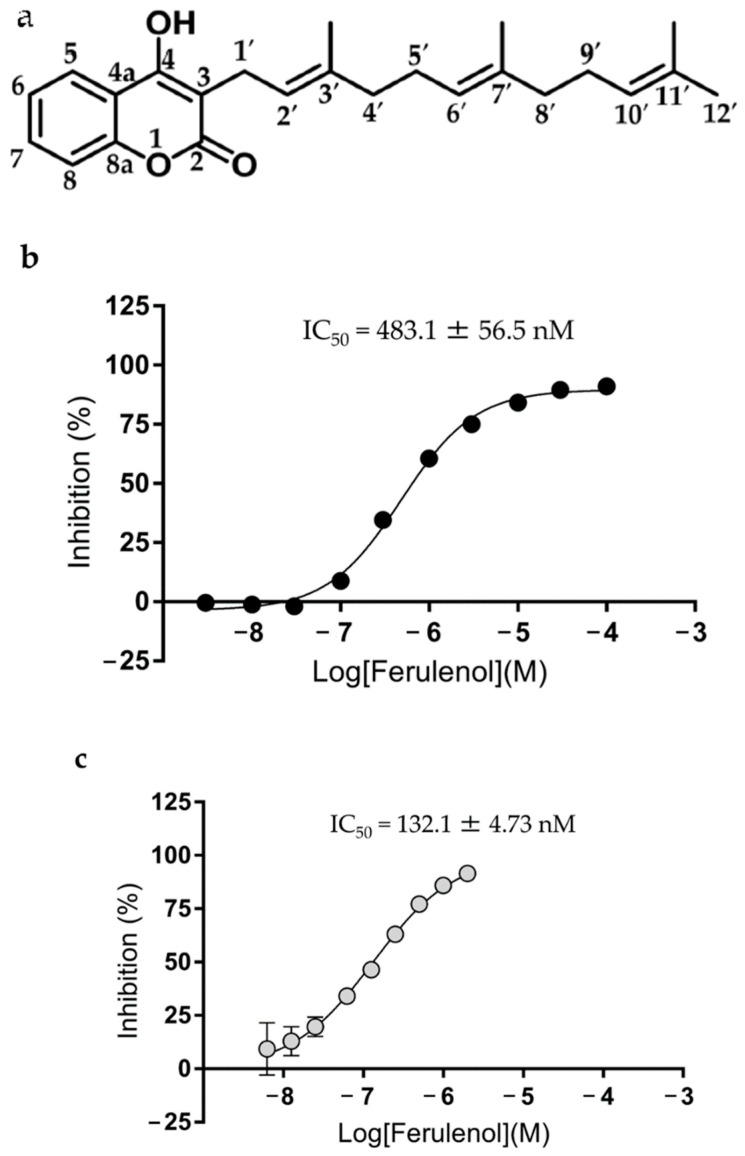
(**a**) Structure of ferulenol. Inhibition curves of ferulenol for EtDHODH (**b**) and HsDHODH (**c**). Values are plotted as mean ± SD (*n* = 4).

**Figure 3:**
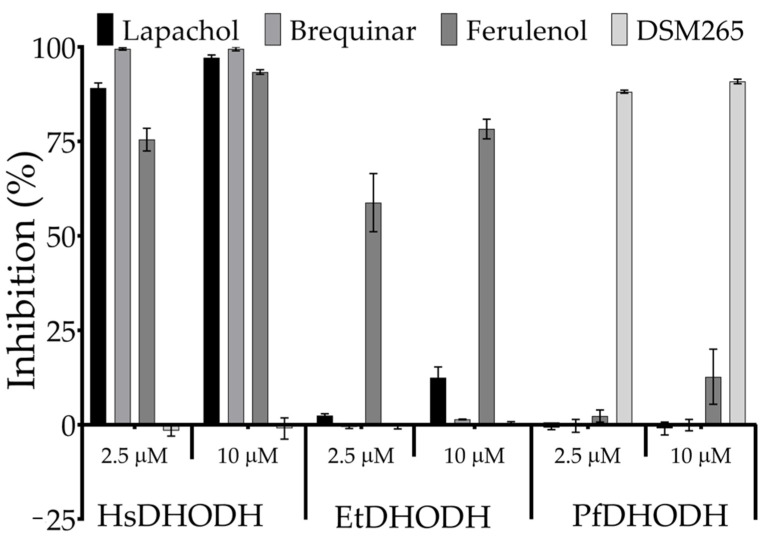
Cross-sensitivity of HsDHODH, EtDHODH, and PfDHODH to lapachol (black), brequinar (dark gray), ferulenol (light gray) and DSM265 (white). Lapachol and brequinar are positive controls for HsDHODH inhibition, whilst ferulenol and DSM265 is the positive control for EtDHODH and PfDHODH, respectively. Values are plotted as mean ± SD (*n* = 4).

We previously identified ferulenol (Figure 2a) as the first potent and non-competitive inhibitor of *P. falciparum* MQO (IC_50_ = 57 nM) (with respect to ubiquinone), and as the first competitive inhibitor of trypanosome alternative oxidase (IC_50_ = 1.42 nM) (with respect to ubiquinol) [41,48]. During our routine search for inhibitors of EtDHODH, we found that ferulenol also is an effective inhibitor of EtDHODH, exhibiting an IC_50_ (mean ± SD) of 483.1 ± 56.5 nM (Figure 2b). Moreover, the evaluation of the species-selectivity of ferulenol (Figure 2c) revealed that this compound also inhibited HsDHODH with an IC_50_ of 132.1 ± 4.73 nM. In contrast, the enzymatic activity of PfDHODH was not inhibited by ferulenol, even at a concentration as high as 20 μM. To test whether EtDHODH shares cross-sensitivity to inhibitors of HsDHODH and PfDHODH, we assayed the effect of lapachol, brequinar, ferulenol and DSM265 on the enzymatic activities of EtDHODH, HsDHODH, and PfDHODH (Figure 3). As expected, lapachol and brequinar potently inhibited HsDHODH. However, neither compound exhibited significant inhibition of EtDHODH and PfDHODH (Figure 3). Similarly, DSM265 showed specific inhibition to PfDHODH. Ferulenol inhibited both of EtDHODH and HsDHODH enzymatic activities. These results indicated that EtDHODH does not share cross-sensitivity to classical DHODHs inhibitors, though ferulenol does inhibit the enzymatic activities of EtDHODH and HsDHODH.

### 3.6. Overall Structure of EtDHODH and Comparison to HsDHODH Structure

**Figure 4:**
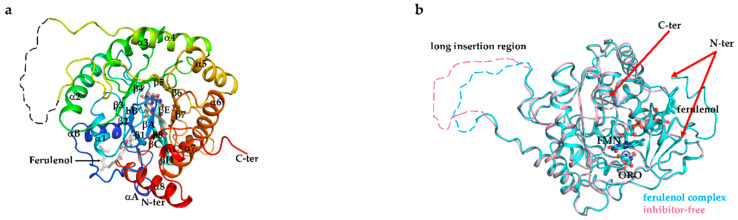
(**a**) Overall structure of EtDHODH. Cartoon model is colored in rainbow from blue (N terminus; N-ter) to red (C terminus; C-ter). The hydrophobic N-terminal domain composed of two α-helices is illustrated in blue (αA and αB). The catalytic central barrel ((α/β)_8_ barrel) is composed of eight parallel β strands (β1–β8) and surrounded by eight α-helices (α1–α8). Another α helices and β strands are labeled αC and βA to βE, respectively. (**b**) Superimposed images of EtDHODH-ferulenol complex and inhibitor-free structures. The dashed dotted lines represent the long insertion loop for which an electron density map was not readily visible. Orotate (ORO), flavin mononucleotide (FMN) and ferulenol molecules are shown in the ball and stick models.

The crystal structures of inhibitor-free EtDHODH and EtDHODH-ferulenol complex were determined at resolutions of 3.5 Å and 3.65 Å, respectively (Figure 4). Both crystal forms contain four EtDHODH chains per asymmetric unit. The fact that; (i) there are no significant intermolecular interactions in either crystal structure, (ii) EtDHODH migrates at similar sizes with PfDHODH and HsDHODH, i.e., around 45 kDa, in high-resolution clear native electrophoresis (Figure S2) using dodeylmaltoside buffer (micelle size of 72 kDa) and visualized by CBB and DHODH activity staining collectively suggest that EtDHODH is functionally monomeric, consistent with other family 2 DHODHs such as those from human and *P. falciparum*. EtDHODH contains two domains connected by an extended loop and corresponding to a small N-terminal domain (N-terminus to Ala31) and a large C-terminal domain (Asp43 to the C-terminus) (Figure 4a). The small N-terminal domain is the enzyme’s membrane binding region, which forms two N-terminal amphiphilic helixes (αA and αB). The large domain has an α/β-barrel fold with a central barrel of eight parallel β strands (β1–β8) surrounded by eight α helices (α1–α8) (Figure 4a). An additional pair of antiparallel β strands, βA and βB, are located at the bottom of the barrel (Figure 4a). The large domain of EtDHODH contains additional antiparallel β strands (βC, βD, and βE) at the top of the barrel (Figure 4a). The long insertion region of EtDHODH (residues 218-243 in the inhibitor-free structure and residues 217-239 in the ferulenol complex structure) is missing from the final structures due to the insertion sequences’ poor electron density (Figure 4a,b).

**Figure 5:**
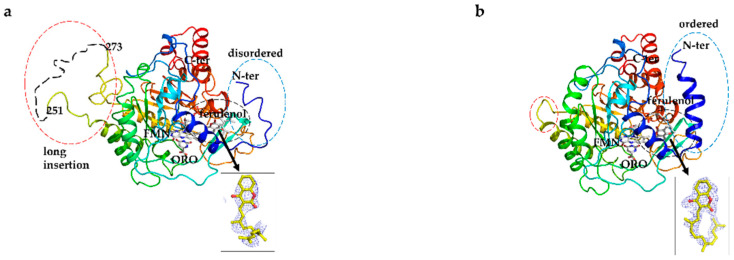
(**a**) Structure of EtDHODH in complex with ferulenol. The extended insertion and N-terminal α helix, seen only in EtDHODH, were disordered, and are highlighted in the dotted red circles. (**b**) Structure of HsDHODH in complex with ferulenol. Note that the long insertion and disordered N-terminal regions (shown in the dotted blue circle) are not found in HsDHODH. The N-terminal V-shaped membrane-associated region is clearly visible in HsDHODH (blue helices) but not in EtDHODH. N-teminus and C-terminus are denoted as N-ter and C-ter, respectively. Ferulenol, flavin mononucleotide (FMN) and orotate (ORO) are highlighted in white ball-and-stick representation. The 2Fo –Fc electron density map (contoured at 1 σ) of bound ferulenol (yellow stick) is highlighted as blue mesh in the right bottom in both figures.

Although, the overall structures of both enzymes were similar, there were two marked differences, which were located at the N-terminal helix region and at the long insertion region. In the ligand-free EtDHODH crystal structure, the electron density map of the N-terminal helix region (αA) was disordered, indicating the high flexibility of this region (Figure 5a). On the other hand, in the HsDHODH-ferulenol complex structure, the N-terminal region was clear and formed an extended α-helix (αA, Figure 5b). Met43 and Leu48, residues located in the extended N-terminal (αA) region of HsDHODH, interact with the isoprenyl tail of ferulenol (Figure 5b). Further, in the HsDHODH-ferulenol structure, two helices (αA and αB) together form a V-shaped, membrane-associated region where the inhibitor is bound. The extended insertion region (35 residues), located between α4 and β5 in EtDHODH (Figure 4a and Figure 5a), and the corresponding region in HsDHODH (Figure 5b), are located far from the active site and may not affect the binding of substrates or inhibitors to either enzyme.

### 3.7. Recognition of Ferulenol by EtDHODH and HsDHODH

**Figure 6:**
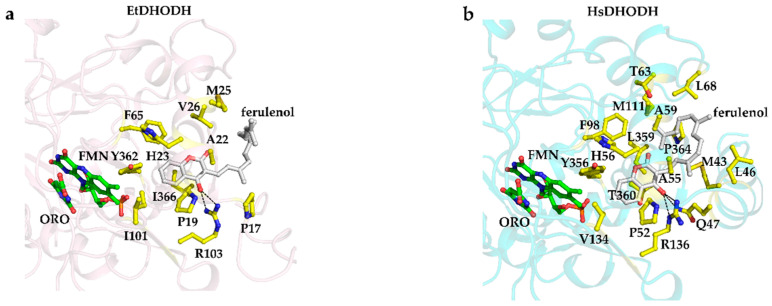
The binding mode of ferulenol (white) in EtDHODH (**a**) and HsDHODH (**b**). Residues located within approximately 4.0 Å of bound ferulenol are shown in yellow ball-and-stick representation. Bound ORO and the cofactor FMN are shown as green ball-and-stick representation. The hydrogen bonds between ferulenol’s O4 oxygen atom and Arg103 are shown as dashed lines.

In the EtDHODH-ferulenol complex crystal structure, the ferulenol molecule was bound to a hydrophobic cavity formed by the N-terminal helices (αA and αB); these N-terminal helices are predicted to be the ubiquinone binding site (Figure 5a). The N-terminal region of EtDHODH-ferulenol complex (LLEVVYGLLPENFP), which is visible in the crystal structure, is stabilized by weak hydrophobic interactions with the His108, Tyr367, Arg368, and Arg375 residues. The aromatic ring of ferulenol is surrounded by Pro19, Ala22, His23, Val26, Phe65, Ile101, Tyr362, and Ile366 to form hydrophobic interactions (Figure 6a). Hydrogen bonds are formed only between the ferulenol O4 oxygen atom and the enzyme’s Arg103 guanidino group, which defines the orientation of the aromatic ring (Figure 6a). The isoprenyl tail of ferulenol is surrounded by EtDHODH hydrophobic residues Pro17, Ala22, Met25, and Val26.

**Figure 7:**
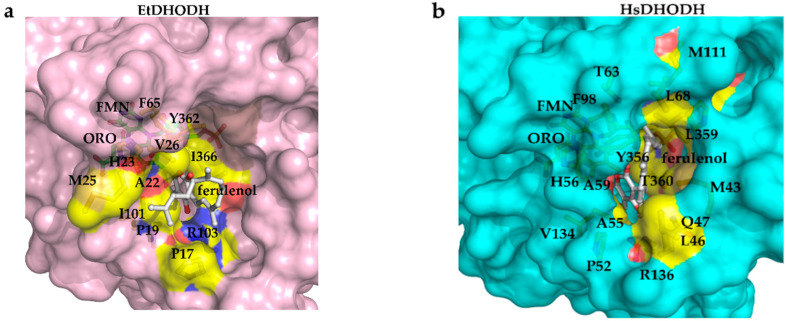
Surface representation showing the differences in the ferulenol (white) binding site between EtDHODH (**a**) and HsDHODH (**b**). Color codes are that same as those described in Figure 6.

From the co-crystal structures, we found that the binding mode of ferulenol is difference between EtDHODH and HsDHODH. The inhibitor is bound in the hydrophobic pocket located between the two N-terminal amphiphilic helixes (αA and αB) in HsDHODH and in an equivalent region of EtDHODH (Figure 6a,b). However, as shown in Figure 6, ferulenol binds deeper in the hydrophobic pocket of HsDHODH compared to EtDHODH. The aromatic ring of ferulenol in the HsDHODH-ferulenol complex is surrounded by 10 residues (Gln47, Pro52, Ala55, His56, Ala59, Phe98, Val134, Arg136, Tyr356, and Thr360), forming many hydrophobic interactions (Figure 6b). The number of residues that are located within 4 Å of the aromatic ring of ferulenol are reduced to 8 (Pro19, Ala22, His23, Val26, Phe65, Ile101, Tyr362, and Ile366) in EtDHODH (Figure 6a,b). For HsDHODH, in addition to Arg136 (corresponding to Arg103 from EtDHODH), the amide nitrogen atom of Gln47 forms an additional hydrogen bond to the O4 oxygen atom of ferulenol (Figure 6b). The isoprenyl tail of ferulenol bound to HsDHODH folds within the hydrophobic pocket and interacts with Met43, Leu46, Gln47, Ala55, Ala59, Thr63, Leu68, Phe98, Met111, Arg136, Leu359, and Pro364 through hydrophobic interactions (Figure 6b). On the other hand, the isoprenyl tail of ferulenol is bound facing the outside environment in EtDHODH with less interaction with amino acid residues. Consequently, the interactions of the isoprenyl tail of ferulenol are clearly tighter in HsDHODH than in EtDHODH (Figure 6 and Figure 7), which could be the reason for the increased inhibition potency towards HsDHODH.

## 4. Discussion

The pyrimidine de novo biosynthesis pathway in apicomplexan parasites is an attractive target for drug development. Amongst the 6 enzymes of the de novo pathway, DHODH is the rate-limiting step and the key enzyme connecting pyrimidine biosynthesis to ETC at the level of ubiquinone. *T. gondii* can obtain pyrimidines through both the de novo and salvage pathways [49]. *E. tenella* has been reported to harbor an active pyrimidine de novo pathway, but to have only a negligible (or no) salvage pathway [19]. Other pathogens such as *P. falciparum* [50] and also *H. pylori* [51] lack the enzymes required for the salvage pathway, making those organisms highly dependent on the de novo pathway. This observation had led to the development of DSM265, a potent inhibitor of PfDHODH that has shown an excellent anti-plasmodial profile in Phase II clinical trials [27]. Separately, we recently reported the discovery of intervenolin and its derivatives as potent and specific *H. pylori* DHODH inhibitors; intervenolin shows excellent antibacterial activity in both in vitro and in vivo models [23]. Other enzyme from nucleotide biosynthesis such as dUTPase (deoxyuridine 5′-triphosphate nucleotidohydrolase, E.C.36.1.23), a house-keeping enzyme that catalyzes the hydrolysis of dUTP to PPi and dUMP, which in turn, is used as precursor dTMP biosynthesis, have been reported to be indispensable for parasite survival within erythrocytes and a druggable target [52,53,54].

Due to the high economic impact of coccidiosis in the poultry industry, we performed the first characterization of the mitochondrial ETC from *E. tenella* sporozoites [55]. At the sporozoite stage, this parasite showed very high activity for enzymes of the ETC, with the exception of DHODH. These data are consistent with the fact that sporozoites are in a non-dividing resting state, implying that the pyrimidine de novo pathway is inactive at this stage, resulting in extremely low DHODH activity. Indeed, previous reports have shown that sporozoites possess a low rate of RNA synthesis, which increased approximately 15-fold in the merozoite stage [20]. Accordingly, the activities of enzymes from the pyrimidine de novo pathway are reportedly low in the lysates of sporozoites [18]. As the *E. tenella* DHODH activity was barely detected in our previous study, we sought to determine whether the recombinant EtDHODH is functionally active. In the present study, we purified EtDHODH with a specific activity of 31 μmol/min/mg protein (*K*_cat_ = 25.8 s^−1^), a value higher than those reported for the *P. falciparum* orthologue [56,57]. Hence, these data provide the first direct evidence that EtDHODH is functionally active and strengthen the hypothesis that the pyrimidine de novo biosynthesis pathway is minimally or not active at the sporozoite stage.

As EtDHODH was not inhibited by established HsDHODH inhibitors, we tested the activity of EtDHODH in the presence of various ETC inhibitors, including rotenone, atpenin A5, antimycin A, flutolanil, ascofuranone, and ferulenol. Among these compounds, ferulenol was one of the potent inhibitor of EtDHODH. Recently, we reported that ferulenol is a potent inhibitor of *P. falciparum* MQO (IC_50_ = 57 nM) [41] and trypanosome alternative oxidase (TAO; IC_50_ = 1.42 nM). The crystal structure of TAO in complex with ferulenol was determined at 2.7 Å resolution [48]. It also has been reported that ferulenol is a weak inhibitor of the rat mitochondrial respiratory enzyme succinate:ubiquinone reductase (SQR, IC_50_ = 17,000 nM) [58] and an inhibitor of rat [59] and bacterial [60] vitamin K epoxide reductases (VKORs), with IC_50_s of 98 and 200 nM, respectively. Therefore, ferulenol inhibits the activity of a variety of ETC enzymes and VKOR and may not be used in mammals. In this study, we revealed that EtDHODH is another target of ferulenol, with an IC_50_ value of 483 nM. Inhibitors of HsDHODH have been shown not to inhibit the DHODH activities of the *P. falciparum* [57] and *E. coli* [61] enzymes; similarly, PfDHODH inhibitors do not inhibit HsDHODH [62]. In the present work, we confirmed that ferulenol has no effect on PfDHODH enzyme activity, although the compound does exhibit potent inhibition of HsDHODH (IC_50_ = 132 nM).

It is well-documented that the cross-sensitivity to inhibitors is very low among DHODHs from several species; this observation has been attributed to differences in the structure of the inhibitor binding pockets. Such differences were explored to design DSM265, a potent and specific PfDHODH inhibitor. Consistent with those results, EtDHODH and PfDHODH showed no cross-sensitivity to known HsDHODH inhibitors such as lapachol and brequinar, neither EtDHODH nor PfDHODH was inhibited by DSM265. Because ferulenol inhibited both of EtDHODH and HsDHODH, but not PfDHODH, we expected that the crystal structure of EtDHODH would reveal similarities between the inhibitor binding pockets of EtDHODH and HsDHODH. Interestingly, the binding mode of ferulenol were different between the two enzymes and the number of residues required to interact with the 4-hydroxycoumarin moiety of ferulenol are larger in HsDHODH than EtDHODH. In addition, interactions formed by the isoprenyl chain showed wider differences between the two enzymes. Collectively, these differences in binding mode of ferulenol could be the reason of its stronger inhibition over HsDHODH, compared to EtDHODH. It is important to note that, while EtDHODH and HsDHODH are both sensitive to ferulenol, potent HsDHODH inhibitors (lapachol, brequinar, ascofuranone and several ascofuranone derivatives) are ineffective against EtDHODH (Figure 3 and Figure S3). In order to reveal the structural determinant(s) for the insensitivity to ferulenol by PfDHODH, we have modelled the two binding modes of ferulenol onto the crystal structure of PfDHODH. Our analysis indicates that independently to the binding mode of ferulenol, the Phe188 from PfDHODH, located at the hydrophobic pocket, causes steric hindrance to the isoprenyl chain from ferulenol which makes its binding unfavorable (Figure S4). Similarly, when DSM265 was modelled onto the hydrophobic pocket of EtDHODH, several steric hindrance between the pentafluorosulfanyl moiety and the side chain from V26, M78 and F75 (Figure S5) were observed. These findings are consistent with the crystal structures, obtained in this study, and strongly suggests that the development of selective EtDHODH inhibitors can be achieved.

## 5. Conclusions

In this manuscript, we described the first recombinant expression system for coccidian DHODH and provided direct evidence that DHODH from *E. tenella* is functionally active. In addition, we identified ferulenol as the first nanomolar inhibitor of EtDHODH. Although, EtDHODH and PfDHODH showed no cross-sensitivity to HsDHODH inhibitors such as lapachol and brequinar, EtDHODH and HsDHODH were both sensitive to ferulenol. Moreover, we presented the first crystal structures of a coccidial DHODH (both ligand-free and bound to ferulenol), together with a structure for the HsDHODH-ferulenol complex. The results presented here showed that the ferulenol inhibits EtDHODH and HsDHODH by displaying different binding modes. These differences in interactions with ferulenol may help explain the differences in the IC_50_ values between EtDHODH and HsDHODH. Future studies should be addressed to understand whether or not ferulenol inhibits the chicken (host) enzyme once the method to obtain recombinant DHODH is established. Hence, our results should not be interpreted to justify the use of ferulenol for prevention and control of coccidiosis by the broilers, but to be exploited to accelerate the structure-based design of drugs targeting specifically coccidian DHODH.

## Supplementary Materials

The following are available online at https://www.mdpi.com/2073-4425/11/12/1468/s1, Table S1. Statistics of data collection and structural refinement for EtDHODH and HsDHODH-ferulenol complex structures. Figure S1. Top: sequence alignment of amino acid sequences of DHODH from Eimeria tenella and other organisms. Figure S2. The purity and oligomeric state of EtDHODH in solution evaluated by SDS-PAGE and HrCNE, respectively. Figure S3. Differences in the sensitivity to ascofuranone derivatives between EtDHODH and HsDHODH. Figure S4. Model structure of ferulenol bound to PfDHODH. Figure S5. Superposed structure of DSM265 bound to PfDHODH and EtDHODH.
