# Supplementary material for: Structural and Biochemical Features of Eimeria tenella Dihydroorotate Dehydrogenase, a Potential Drug Target
Source: Genes (Basel). 2020 Dec 7;11(12):1468. doi: 10.3390/genes11121468 (PMC7762340; doi:10.3390/genes11121468)
Supplement: Supplementary file 1 [file genes-11-01468-s001.pdf]

genes MDPI

Table S1. Statistics of data collection and structural refinement for EtDHODH and HsDHODH-ferulenol complex structures

	EtDHODH (ligand-free form)	EtDHODH (ferulenol complex)	HsDHODH (ferulenol complex)
<u>**Data collection**</u>			
Space group	*I*23	$P6_3$	$P3_221$
Cell parameters			
a, *b*, *c* (Å)	248.8, 248.8, 248.8	133.0, 133.0, 215.0	90.2, 90.2, 123.3
α β γ (°)	90,0, 90.0, 90.0	90,0, 90.0, 120.0	90,0, 90.0, 120.0
X-ray source	SPring-8 BL44XU	SPring-8 BL44XU	KEK-PF BL17A
Wavelength (Å)	0.90000	0.90000	0.98000
Resolution (Å)	50-3.5 (3.56-3.5)	50-3.65 (3.71-3.65)	50-1.90 (1.93-1.90)
Total No. of reflections	283,621	100,597	348,775
No. of unique reflections	32,456	24,158	46,303
R_{merge}	0.132 (0.792)	0.075 (0.797)	0.081 (0.729)
R_{means}	0.140 (0.849)	0.085 (0.908)	0.085 (0.783)
$I/\sigma(I)$	5.6 (2.6)	7.9 (1.7)	9.5 (3.0)
Completeness (%)	100.0 (100.0)	96.1 (96.7)	100.0 (100.0)
Redundancy	9.3 (8.3)	4.3 (4.2)	7.5 (7.6)
<u>**Refinement**</u>			
Resolution (Å)	20-3.5	20-3.65	20-1.90
R_{work} / R_{free}	0.272 / 0.359	0.221 / 0.303	0.163 / 0.178
Used reflections	29,340	21,056	43,740
No of non-hydrogen atoms			
Protein	11,285	11,764	2,823
Ligand	168	276	69
Solvent	-	-	159
Average *B*-factors (Å^2)			
Protein	48.0	62.4	29.6
Ligand	34.1 (FMN, ORO)	50.7 (FMN, ORO, ferulenol)	31.1 (FMN, ORO, ferulenol)
Solvent	-	-	36.1
RMSD			
Bond length (Å)	0.013	0.009	0.009
Bond angle (°)	1.796	1.522	1.539
Ramachandran plot (%)			
Favored regions	78.8	79.9	93.4
Allowed regions	20.0	19.1	6.6
Outers	1.2	1.0	0
PDB code	6AJ5	6AJE	6IDJ

Values in parentheses are for the highest resolution shell.

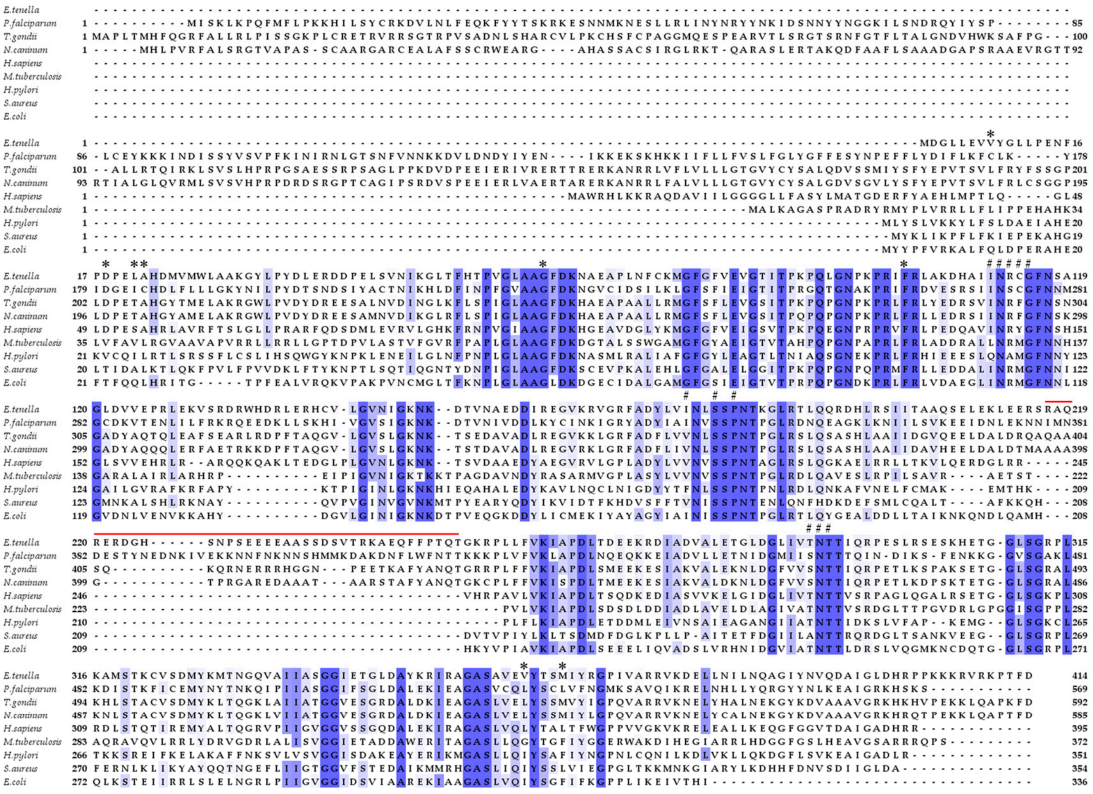

	E. tenella	*P. falciparum*	*T. gondii*	*N. caninum*	*H. sapiens*	*M. tuberculosis*	*H. pylori*	*S. aureus*	*E. coli*
E. tenella		41.56	52.17	52.42	45.52	36.06	32.43	33.25	35.70
P. falciparum			32.93	31.80	34.23	28.24	29.30	28.12	36.95
T. gondii				72.13	43.65	33.41	32.09	33.83	36.32
N. caninum					42.59	33.01	31.36	32.18	35.88
H. sapiens						40.63	36.14	35.77	41.62
M. tuberculosis							38.87	34.08	42.98
H. pylori								34.55	41.23
S. aureus									36.18
E. coli									

Figure S1. Top: sequence alignment of amino acid sequences of DHODH from *E. tenella* and other organisms. Residues were colored according to percentage identity by Jalview. Conserved residues involved in the binding of orotate and coumarin group of ferulenol, according to the crystal structure of *H. sapiens* DHODH (PDB: 6IDJ), are shown with "#" and "*" symbols, respectively. The long insertion (R217 to T252) found only in apicomplexan DHODHs is highlighted in red.

Bottom: Amino acid sequence identity. Abbreviations; *E. tenella*: *Eimeria tenella* (XP013227840), *P. falciparum*: *Plasmodium falciparum* (AAC37170), *T. gondii*: *Toxoplasma gondii* (AAM46067), *N. caninum*: *Neospora caninum* (XP003880770), *H. sapiens*: *Homo sapiens* (AAH65245), *M. tubercu*: *Mycobacterium tuberculosis* (KDA15626), *H. pylori*: *Helicobacter pylori* (ADZ49526), *S. aureus*: *Staphylococcus aureus* (SBB47760), *E. coli*; *Escherichia coli* (KIG45581). All the numbers in the parentheses are GenBank Accession Nos.

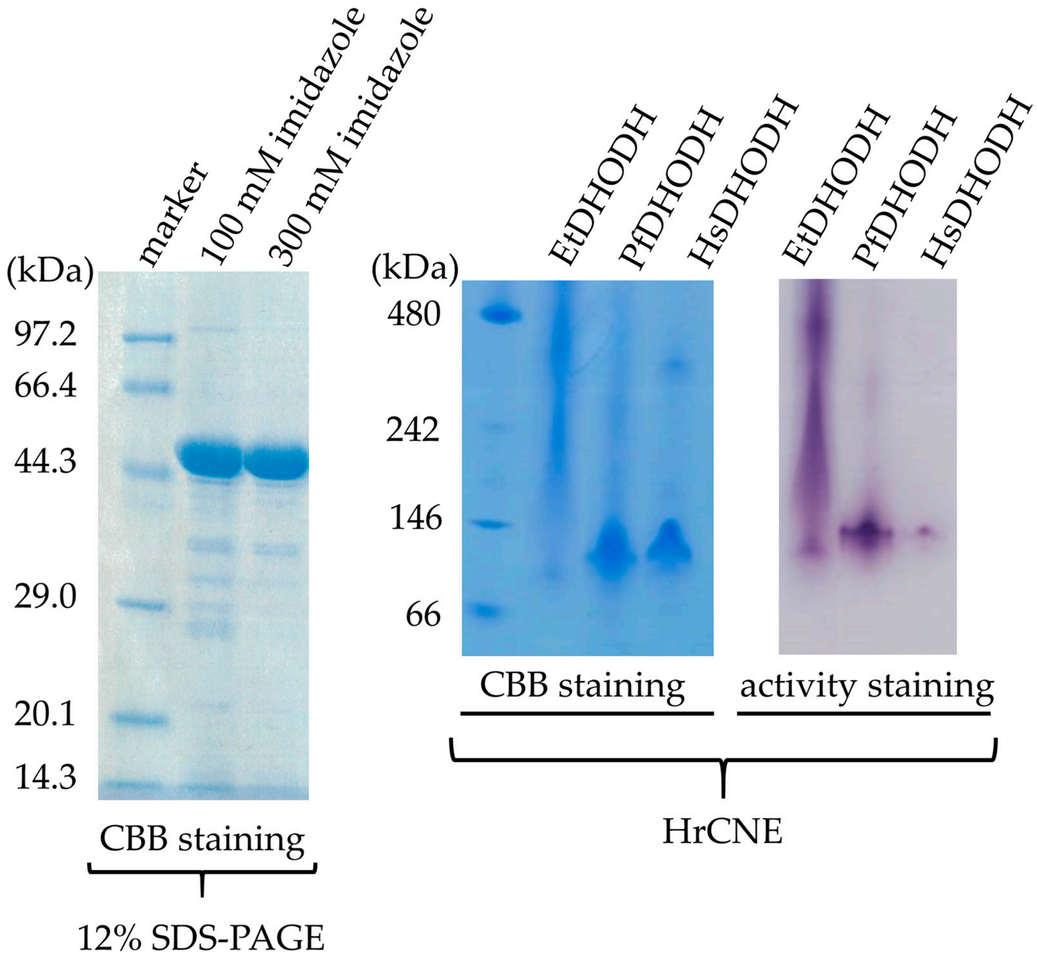

Figure S2. The purity and oligomeric state of EtDHODH in solution evaluated by SDS-PAGE and HrCNE, respectively. The enzyme eluted by 100 mM and 300 mM imidazole was loaded (5 µg) onto SDS-PAGE (12%) and stained by CBB (left panel). The fraction eluted at 300 mM was used for subsequent studies. The oligomeric state of EtDHODH in solution was evaluated together with PfDHODH and HsDHODH by high-resolution clear native electrophoresis (HrCNE), followed by CBB and DHODH activity staining (right panel), which were performed essentially as previously reported (see reference 22 from the main text). The calculated molecular weight (MW) from the amino acid sequences are 46 kDa, 47 kDa and 40 kDa for EtDHODH, PfDHODH and HsDHODH, respectively. The buffer used for HrCNE contained dodecylmaltoside (DDM) which has a micelle size of 72 kDa and cause the shift in MW of the three enzymes to 110-130 kDa. Small variations in the size of the three enzymes are characteristic of a globular protein. Altogether, these results indicate that the three enzymes are active as monomer.

Figure S3

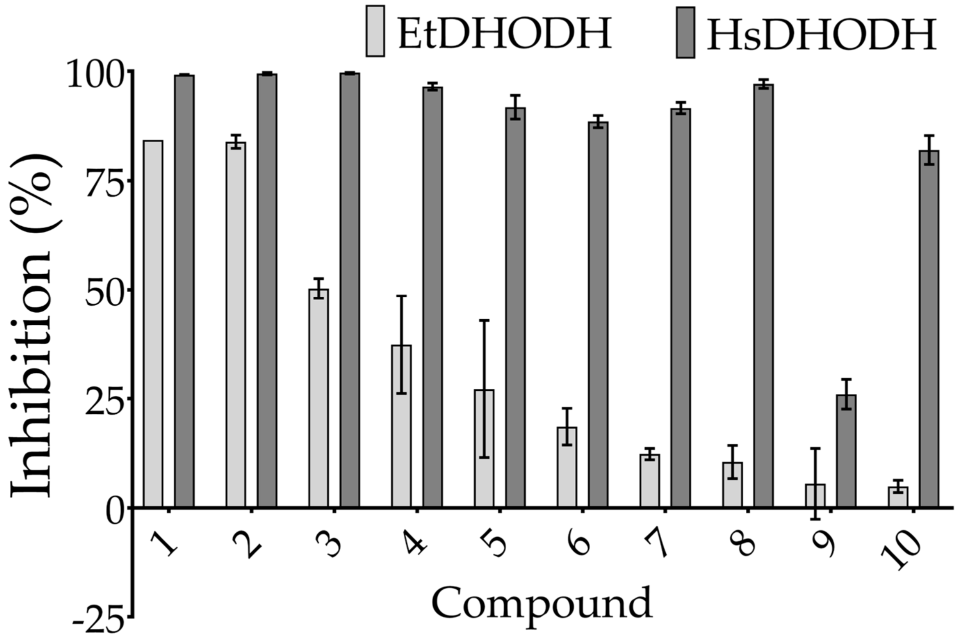

1

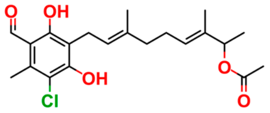

2

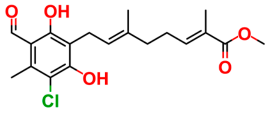

3

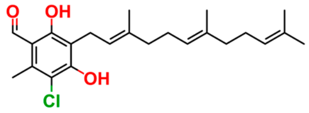

4

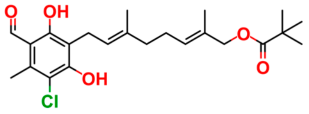

5

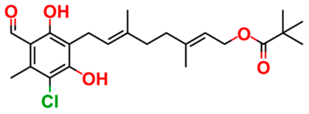

6

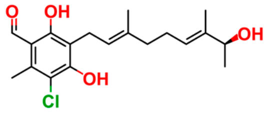

7

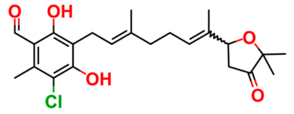

8

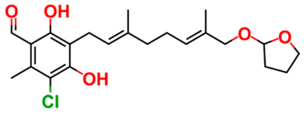

9

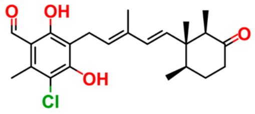

10

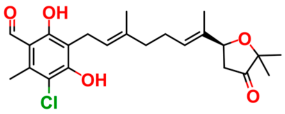

Figure S3. Differences in the sensitivity to ascofuranone derivatives between EtDHODH and HsDHODH. The inhibition of each compound against EtDHODH (dark gray) and HsDHODH (light gray) at 2.5 μM are shown as the average. The error bars represent the SD (n = 4). The structure of the compounds selected for this figure (compounds **1-10**) is shown at the bottom.

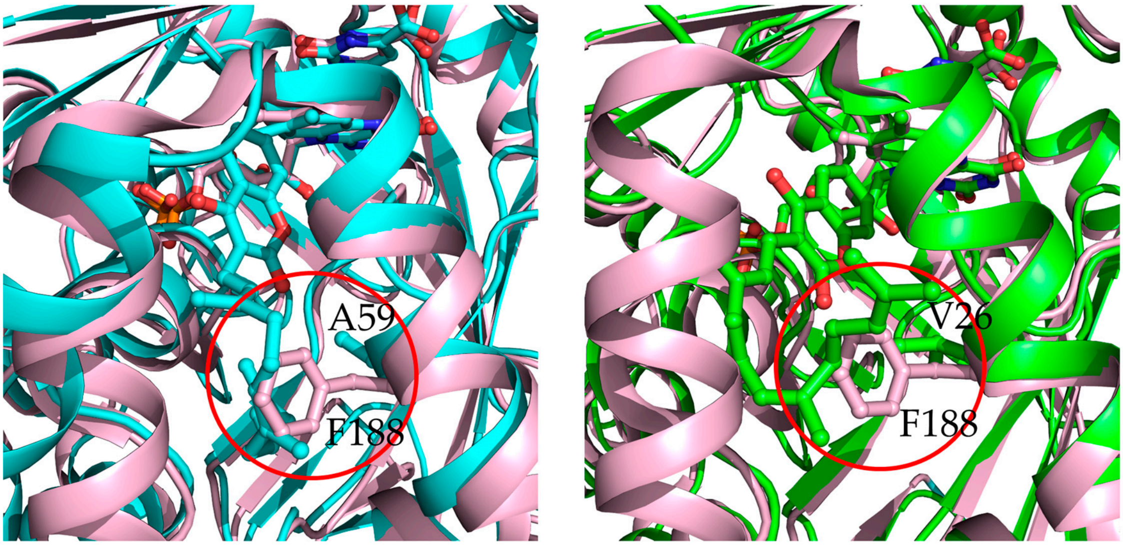

HsDHODH-Ferulenol: cyan
PfDHODH-Ferulenol (model): pink

EtDHODH-Ferulenol: green
PfDHODH-Ferulenol (model): pink

Figure S4. Model structure of ferulenol bound to PfDHODH. Binding of ferulenol to PfDHODH is not favorable due to steric hindrance of isoprenyl chain with F188 (red circle). The F188 from PfDHODH is replaced by A59 and V26 in HsDHODH and EtDHODH, respectively. Structure, residues and ligands shown in cyan, pink and green are from HsDHODH, PfDHODH and EtDHODH, respectively.

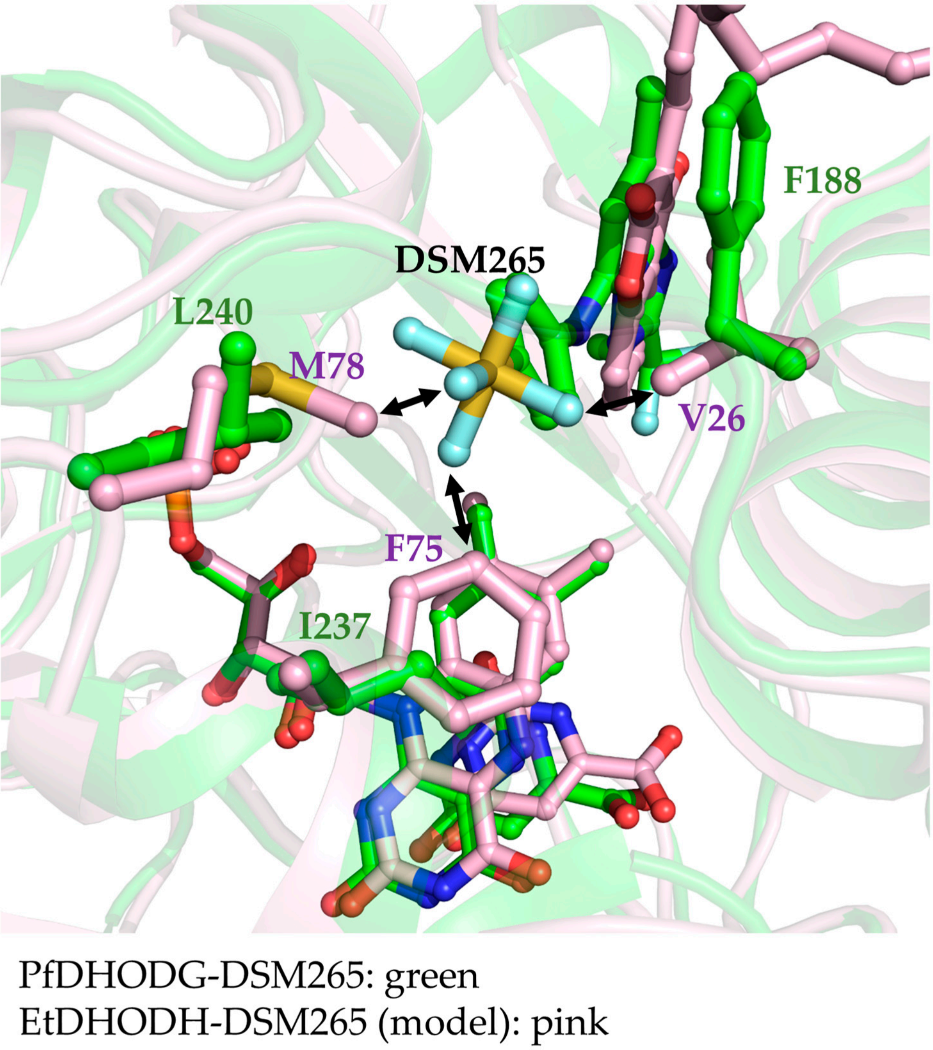

Figure S5. Superposed structure of DSM265 bound to PfDHODH and EtDHODH. Binding of DSM265 from PfDHODH (4RX0) was modelled into EtDHODH, which show several steric hindrance with the pentafluorosulfanyl moiety and side chain from V26, M78 and F75 in EtDHODH. Ligands and residues from PfDHODH and EtDHODH are shown in green and pink colors, respectively.
